# Leveraging Visual Place Recognition to Improve Indoor Positioning with Limited Availability of WiFi Scans

**DOI:** 10.3390/s19173657

**Published:** 2019-08-22

**Authors:** Michał R. Nowicki, Piotr Skrzypczyński

**Affiliations:** Institute of Control, Robotics and Information Engineering, Poznan University of Technology, 60-965 Poznan, Poland

**Keywords:** indoor positioning, graph-based optimization, WiFi fingerprinting, visual place recognition

## Abstract

WiFi-based fingerprinting is promising for practical indoor localization with smartphones because this technique provides absolute estimates of the current position, while the WiFi infrastructure is ubiquitous in the majority of indoor environments. However, the application of WiFi fingerprinting for positioning requires pre-surveyed signal maps and is getting more restricted in the recent generation of smartphones due to changes in security policies. Therefore, we sought new sources of information that can be fused into the existing indoor positioning framework, helping users to pinpoint their position, even with a relatively low-quality, sparse WiFi signal map. In this paper, we demonstrate that such information can be derived from the recognition of camera images. We present a way of transforming qualitative information of image similarity into quantitative constraints that are then fused into the graph-based optimization framework for positioning together with typical pedestrian dead reckoning (PDR) and WiFi fingerprinting constraints. Performance of the improved indoor positioning system is evaluated on different user trajectories logged inside an office building at our University campus. The results demonstrate that introducing additional sensing modality into the positioning system makes it possible to increase accuracy and simultaneously reduce the dependence on the quality of the pre-surveyed WiFi map and the WiFi measurements at run-time.

## 1. Introduction

GPS (Global Positioning System) revolutionized the way we navigate outdoors as it is used by pedestrians, cars, planes, and military vehicles to reach certain goals. However, we look for other technologies that would make it possible to obtain accurate position estimates inside buildings because GPS is mostly not available indoors. The availability of accurate position estimates indoors could influence the way people reach their destinations and plan their paths in large buildings. Possible applications include ubiquitous, location-aware advertisement, efficient guidance in public places like train stations and airports, and last but not least, safer and faster evacuation from buildings in case of emergency.

Among the available solutions, the most desirable are those that can be used by everybody in a large variety of locations and those that do not require the deployment of any dedicated infrastructure. Infrastructure-free positioning systems are considered cheaper, as no additional hardware like beacons is required, which reduces the costs of deployment, particularly in large environments. Also, positioning systems that do not require users to sport any additional equipment but work with regular smartphones have a much bigger chance for commercial success. These requirements are met by approaches to positioning that utilize sensors commonly available in smartphones and natural localization cues or signals emitted by devices already deployed in the environment for other purposes.

The majority of existing smartphone-based indoor positioning systems employ the mobile device’s sensors to perform multi-sensor localization based on Bluetooth Low Energy (BLE), WiFi signals or magnetic field changes [1]. Inertial sensors present in all modern smartphones are used to estimate the user’s motion enabling continuous position tracking [2]. Despite a large number of existing approaches, a single dominant method that would take the indoor localization market similarly to the GPS still does not exist. Each of the used sensing modalities and proposed approaches has its shortcomings; for example, the magnetic field-based approach relies on magnetometers that are only available in selected models of smartphones, and BLE approaches require additional beacons, whereas WiFi signals could not be captured by third-party applications on Apple’s mobile devices. Reports from indoor positioning competitions, such as the 2016 International Conference on Indoor Positioning and Indoor Navigation (IPIN) [3], suggest that the most commonly deployed positioning systems in smartphone-based and infrastructure-free competition were based on Android devices and utilized multi-sensor fusion based on WiFi scanning, inertial sensing, and additional, custom processing strategies, e.g., detecting when a person is turning. Nevertheless, the recent changes in Android WiFi scanning policy (https://developer.android.com/guide/topics/connectivity/wifi-scan) will require modifications to those approaches since the WiFi scanning rate is reduced to four scans per two minutes in the latest Android devices. This is a significantly lower value than the scanning frequency typically used to obtain a reasonably dense positioning with WiFi-based fingerprinting [4].

Therefore, we propose an indoor positioning system that utilizes typical data from the sensors of a mobile device, such as WiFi scans, inertial measurements, and orientation information, but fuses these data with qualitative information coming from place recognition based on images. Qualitative information is usually ignored in positioning fusion schemes due to challenges with proper representation of the measurements. In our approach, the fusion is performed as graph-based optimization, which is the state-of-the-art solution for Visual Odometry and Simultaneous Localization and Mapping in robotics [5].

The concept of localization constraints stemming from qualitative observations of the environment or information entered directly by the user was introduced in our conference paper [6]. However, this journal article significantly extends the preliminary approach from [6], building upon our more recent graph-based positioning system utilizing WiFi fingerprinting [4]. This paper introduced an efficient opportunistic WiFi sensing framework that does not require laborious surveying of the site, which is also adopted in the research presented here. The novel contribution of this article can be summarized as:

- We introduce a new variant of our visual place recognition (VPR) algorithm that provides accurate user position even in a self-similar environment with multiple corridors.
- We describe in details how to integrate VPR from image sequences in the graph-based position estimation framework.
- We demonstrate experimentally that the proposed approach can be applied even when the WiFi scans are available less frequently, making the WiFi map incomplete, and rendering the absolute position updates from WiFi-based fingerprinting too sparse for correct trajectory estimation. Thus, we show that our new approach meets the needs arising from the recent changes in Android security policies.

## 2. Related Work

Even though indoor positioning with mobile devices is a very active field of research with potentially high market value, none of the approaches proposed so far can be considered dominant. Infrastructure-free positioning systems offer better chances for wide adoption than the iBeacon technology [7,8] due to much lower deployment costs and better scalability with respect to the size of the environment. The indoor positioning competition at the IPIN conference, e.g., in 2016 [3] and 2017 [9], brings teams with different ideas about how the indoor localization should be performed. Similar infrastructure-free competition, like the Microsoft Indoor Localization Competition during IPSN conferences, reveal similar trends because most of these solutions employ the smartphone’s internal sensors (accelerometers, gyroscopes, magnetometers) to obtain data for a pedestrian dead reckoning (PDR) module, which is then combined with less frequent absolute localization information computed by processing WiFi and/or Bluetooth signals. If a single type of sensor is used, like PDR in the winning solution [10] at the 2018 Microsoft Indoor Localization Competition, there is no need for additional fusion of information from multiple sources. This was an exception, as most commonly, there are multiple sources of information and particle filtering is applied as the fusion and estimation framework, as in [11], because particle filters make it possible to model multi-modal distribution, and thus can represent localization ambiguity. Although this is not possible with the Kalman filter [12], this framework is also applied to indoor positioning due to its computational efficiency [13]. Some of the state-of-the-art positioning systems combine one of the basic approaches with additional ideas that make them more robust and accurate, i.e., by employing a graph of possible routes in the environment [14]. Also, information from known environment maps (floor plans) can be used to constrain the localization estimates, which can easily be done when particle filtering is used [15], but is more problematic in the case of Kalman filtering [16].

If an infrastructure-free system is considered, the most used information source is the WiFi adapter. Two basic approaches to WiFi-based positioning are described in the literature: multilateration and fingerprinting. The former estimates the distances between the Access Point (AP) locations and the receiving mobile device using the strength of the received signals and some model of the WiFi signal propagation [17]. The latter approach avoids direct modeling of signal propagation by defining the fingerprints—vectors that contain the information about the AP (network ID) and the received signal strength for the current position of the receiver [18]. Fingerprints are surveyed for a large number of positions in a given location during the off-line phase. The collected fingerprints form a WiFi map that is then used online to localize the smartphone. Different methods for matching of the fingerprints and position determination have been proposed in the literature. Probabilistic fingerprinting models the fingerprints as the Gaussian probability density function [19]. Although the probabilistic formulation can improve the positioning accuracy, and it was demonstrated to work on different smartphones [20], it increases the computing power requirements in the mobile device. Thus, it was also proposed in a variant that is able to off-load computations to other devices in the so-called fog computing concept [21]. However, if all computations are accomplished on the mobile device, as in [22], there is no need to use Internet access all the time, which can be an important advantage to the users. Systems lacking any centralized processing are also more scalable with respect to the number of users and more robust to hardware failures. Therefore, our indoor positioning system uses only local processing. We have chosen a deterministic variant of fingerprinting that takes advantage of quickly computable similarity metric, and the *k*-Nearest Neighbor [23] method, which is arguably the most widely investigated WiFi-based positioning technique. In a large environment surveying Surveying the WiFi map in a large environment becomes an issue. The density of a WiFi map can be increased without anadditional amount of time and labor required for physical surveying by leveraging a signal propagation model for the generation of additional “virtual” fingerprints [24]. An alternative solution, which does not need to assume any propagation model is crowdsourcing—updating the WiFi map with data collected by many users while they use the positioning service. This approach was investigated and shown feasible in our recent work [4], and can be adopted also and can also be adopted to the solution proposed in this article. However, our aim in this research is not to improve the efficiency of the off-line preparation phase, but to find a solution that can compensate for the problem with WiFi signal availability during the localization phase that emerged due to the security policy changes in the recent generation of smartphones. Thus, our problem is not that producing a dense WiFi map takes time and labour, but that the localization with WiFi becomes hardly possible with the recent variants of Android OS. While other measurable signals can be potentially used for infrastructure-free indoor positioning, such as anomalies of the ambient magnetic field [25], and even light sources [26], none of these solutions gained popularity so far as they struggle with serious calibration and reliability issues. Moreover, some smartphones have a limited set of sensors, e.g., the magnetometer is not available in many cheaper models.

Teams competing in the indoor positioning challenges mostly use modern smartphones equipped with high-resolution cameras but do not utilize these sensors. Indeed, using vision for smartphone-based positioning requires some user cooperation and does not provide an exact metric estimate of the user’s pose (monocular vision), while image processing on a mobile device requires a lot of computing power [27]. Nevertheless, there are solutions like [28], that try to utilize the camera for indoor localization. More work on VPR can be found in robotics [29]. The seminal paper on FAB-MAP [30] gave rise to a series of research focused on efficient image representations and matching strategies, such as Bags of Visual Words [31] used for place recognition with the detected and described local visual features. The computational efficiency of this approach was improved in [32], where authors utilized binary features, making it possible to operate in larger environments. Even though recent vision-based place recognition methods are fast and robust, they provide only topological information: from the stored images they return the one, which is most similar to the query (i.e., current scene view). Unfortunately, no metric information is made available as the two images might be taken from different distances and viewing angles. Another possibility is to process sequences of images, as proposed in SeqSLAM [33]. Considering entire sequences makes it possible to correctly localize the camera even when individual images do not provide enough information, i.e., due to self-similarities in the environment. This idea was extended in [34] utilizing a single global Local Difference Binary (LDB) descriptor per image. We have shown in our previous work [35] that this method can be implemented in a smartphone, presenting FastABLE—a significantly faster version of the ABLE-M algorithm from [34].

Graph-based optimization is a state-of-the-art alternative to the filtration algorithms in robotics and computer vision. Measurements taken by the sensory system are represented as edges that constrain the graph vertices that represent user’s positions. The graph is then optimized to determine the most probable locations of vertices that jointly explain all constraints. Although graph-based optimization does not maintain several location hypotheses, like a particle filter, it does not marginalize the past states (positions) of the moving agent, like a Kalman filter. Thus, a whole agent trajectory is estimated, and it can be re-computed upon request if new measurements (constraints) become available. This approach gained popularity in robotics [5] due to the efficient solvers, like g${}^{2}$o [36], that make it possible to simultaneously optimize hundreds of parameters with thousands of constraints in real time. The graph-based approach to indoor positioning was introduced in our previous research concerning the localization of single [37] and multiple users [4]. In these papers, we focused on the positioning accuracy of a system based on metric measurements—WiFi and inertial. In contrast, our paper [6] focused on determining additional localization constraints from qualitative visual observations and direct user input, but only a brief feasibility study was presented without thorough evaluation. In this article, we extend the previous research by completely reworking the VPR, as it no longer requires user input and is based on sequences of images, like in [35].

## 3. Graph-Based Optimization for Personal Indoor Localization

The problem considered in this paper is how to combine the user trajectory information obtained from several sources of very different characteristics with respect to the quality of the position estimates and the frequency this information can be obtained. We deal with two types of information: the PDR system provides a semi-continuous, but quite uncertain estimate of the motion, while the WiFi fingerprinting and VPR methods both make it possible to detect already known places. Both types of information define constraints to the current location of the user. To make these constraints useful for positioning we employ a graph-based model, which allows optimization of the collected constraints (measurements) to determine the best location estimate. The graph of constraints is a state-of-the-art representation of optimization problems in robotics due to the common availability of fast solvers like g${}^{2}$o [36] or GTSAM [38]. In the graph, the nodes represent the parameters that are optimized while measurements correspond to the edges that can join one or multiple nodes depending on the type of measurement.

**Figure 1:**
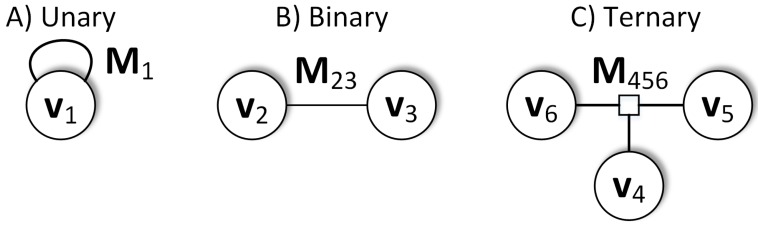
The typical representation of the constraints used in graph-based optimization. An edge joining a single location with measurement is called unary (**A**), joining two locations with measurement is called binary (**B**), and ternary (**C**) is the case of joining three nodes with a measurement. It is also possible to create a constraint joining more nodes, but it rarely occurs in practice.

Let us consider a measurement $M_{i,j,\cdots ,n}$ represented as an edge that joins multiple optimized parameters ($v_{1}$, $v_{2}$, ..., $v_{n}$). The error for that edge can be defined as:

(1) $$e_{i,j,\cdots ,n}\overset{\mathrm{def}}{=}e\left(v_{1},v_{2},\cdots ,v_{n},M_{i,j,\cdots ,n}\right),$$

where $e_{i,j,\cdots ,n}$ can be an arbitrary vector function of the input variables defined in (1), although to obtain good optimization results this function should have a continuous derivative. The edge joining only a single node is called unary (Figure 1A), joining two nodes is called binary (Figure 1B) and joining three nodes is called ternary (Figure 1C). Each edge has a corresponding symmetric square information matrix Ω of the dimensions compatible with the dimensionality of the error vector $e_{i,j,\cdots ,n}$. The information matrix is the inverse of the covariance matrix of the measurement and can be determined experimentally or derived from prior knowledge about the uncertainty of the sensor measurements (an example procedure for RGB-D measurements is given in [39]). In practice, it is often considered as a weight of the measurement importance and it is chosen directly upon the analysis of preliminary optimization results.

Now let us consider the set E of all edges that are introduced into the current graph-based optimization:

(2) $$\underset{e}{⋀}e\in E,$$

where e are individual edges (i.e., constraints). The edges that belong to this set define our optimization problem. The graph-based optimization framework minimizes the weighted square error of every edge in the graph, which can be written as:

(3) $$\underset{v_{1},v_{2},\cdots ,v_{n}}{\mathrm{argmin}}\;E=\sum_{i=1}^{h}e_{i}^{T}\Omega_{i}e_{i},$$

where $e_{i}$ is the *i*-th edge/measurement in the graph out of the total *h* edges in E, and $\Omega_{i}$ stands for the information matrix that corresponds to the *i*-th measurement. The system is optimized using a gradient-based algorithm to determine the most likely configuration of nodes (values), which is an equivalent of the optimization of the function *E*. As a result, the most plausible sequence of the nodes ($v_{1}$, $v_{2}$, ..., $v_{n}$) is found that provides the best explanation of the *h* existing measurements in the graph. Since the optimization is performed with a gradient-based method [36], the process converges to a local minimum. A poor initial guess of the location of nodes may result in a solution that is significantly worse than the global minimum, which makes the choice of the initial configuration of the graph important.

**Figure 2:**
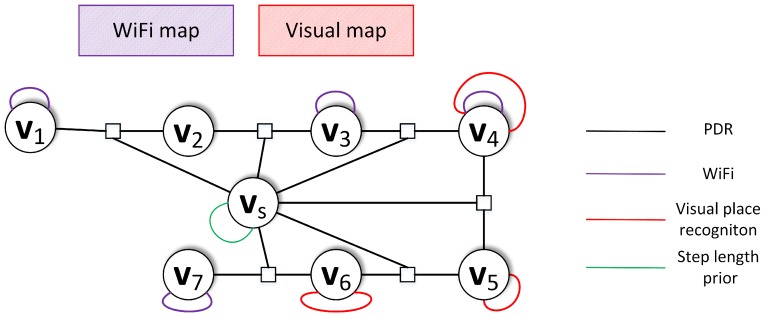
Exemplary graph containing measurements from pedestrian dead reckoning (PDR), WiFi Weighted K Nearest Neighbors (WKNN), visual place recognition (VPR), and step length prior placed on the step estimation node.

The proposed system focuses on utilizing all available information to determine the most probable localization estimate. Therefore, the metric and non-metric sensor measurements are represented as the edges in the graph-based optimization (Figure 2). The metric edges include information coming from PDR and WiFi localization. The non-metric edges extend the already included information by introducing information coming from VPR. Each edge has a corresponding error function and information matrix that captures the importance of the measurement to the localization process. The following sections introduce that information in more details.

## 4. Metric Constraints in the Graph-Based Optimization

### 4.1. PDR

The PDR is a system that determines the current location estimate based on the previous location, the distance traveled, and the information about the change in the direction of walking. The distance is estimated by the stepometer that is implemented based on the Fast Fourier Transform (FFT) of the moving window of the samples consisting of total measured acceleration in all axes [37]. The orientation estimation is performed with the Adaptive Extended Kalman Filter that combines the measurements from accelerometer, gyroscope, and magnetometer [40].

The PDR measurement in the graph-based optimization joins three nodes—previous pose ($v_{i}=\left(x_{i},y_{i},\theta_{i}\right)$), current pose ($v_{j}=\left(x_{j},y_{j},\theta_{j}\right)$) and the step length estimate ($v_{s}=s$). With the information about the measurement $M_{ijs}=({\mathrm{step}}_{ij},\Delta \theta_{ij}$) it is possible to define the error of the edge as:

(4) $$e_{ijs}=\left[\begin{array}{c} x_{j}-x_{i}-s\cdot {\mathrm{step}}_{ij}\cdot \cos\left(\theta_{i}+\frac{\Delta \theta_{ij}}{2}\right)\\ y_{j}-y_{i}-s\cdot {\mathrm{step}}_{ij}\cdot \sin\left(\theta_{i}+\frac{\Delta \theta_{ij}}{2}\right)\\ \theta_{j}-\theta_{i}-\Delta \theta_{ij} \end{array}\right].$$

The step length is optimized jointly with the rest of the measurements as presented in our previous work [4]. The information matrix for the PDR edge properly weights the metric and orientation error and is defined as:

(5) $$\Omega^{\mathrm{pdr}}=\left[\begin{array}{ccc} 1 & 0 & 0\\ 0 & 1 & 0\\ 0 & 0 & k^{\mathrm{pdr}} \end{array}\right],$$

where the $k^{\mathrm{pdr}}$ value should be set to $k^{\mathrm{pdr}}\geq 10$ in order to reduce the orientation errors, as these errors have the most deteriorating impact on the final estimate of the user position [37].

### 4.2. WiFi Fingerprinting

The WiFi fingerprinting technique computes the location of the WiFi receiver (in our case a smartphone) in the environment by comparing the currently acquired WiFi scan (the observation) to a map of WiFi scans stored prior to the operation of the positioning system. In the proposed solution, we utilize the Weighted K Nearest Neighbors (WKNN) [41] algorithm that determines the *k* most similar WiFi scans from the database to the currently captured WiFi scan. The similarity measure is defined as the inverse distance between scans:

(6) $$s\left(X,Y\right)=\frac{1}{d_{\mathrm{wifi}}\left(X,Y\right)+eps},$$

where X and Y are the WiFi scans, $d_{\mathrm{wifi}}\left(\cdot ,\cdot \right)$ is the chosen distance measure between scans comprising of unique network identifiers and corresponding signal strength measurements and eps is an infinitesimal value to avoid division by zero. The $d_{\mathrm{wifi}}\left(\cdot ,\cdot \right)$ is usually chosen depending on the type of the environment, the density of the map and the used device from a list of typical measures, like $\ell_{2}$ norm, with more options described and analyzed in [42]. During the localization, the system computes the final position estimate for scan X based on *k* most similar scans from the database based on (6):

(7) $$p_{\mathrm{wifi}}=\frac{\sum_{i=1}^{k}s\left(X,Y_{i}\right)p_{i}}{\sum_{i=1}^{k}s\left(X,Y_{i}\right)},$$

where $p_{i}$ is the position of the *i*-th WiFi scan and $p_{\mathrm{wifi}}$ is the final estimate of the user position.

The information from the WiFi system is added to the graph-based optimization as an edge that constrains the *i*-th user position $v_{i}=\left(x_{i},y_{i},\theta_{i}\right)$. Assuming that the WKNN estimate is equal to $p_{\mathrm{wifi}}=\left(x_{\mathrm{wifi}},y_{\mathrm{wifi}}\right)$, the error of the edge can be written as:

(8) $$e_{i}^{\mathrm{wifi}}=\left[\begin{array}{c} x_{i}-x_{\mathrm{wifi}}\\ y_{i}-\mathrm{wifi} \end{array}\right].$$

The information matrix corresponding to the edge is set to $\Omega^{\mathrm{wifi}}=k^{\mathrm{wifi}}I_{2\times 2}$, where $k_{\mathrm{wifi}}$ is the weight assigned to the WiFi estimate. Currently, this value is assumed to be 10, as in [4].

The WKNN system requires capturing the map of WiFi scans in the environment in selected locations with more locations resulting in more accurate localization estimate. Moreover, parameter tuning is recommended to determine the distance measure $d_{\mathrm{wifi}}$ used to compare scans similarity. In the tests described in this article, we re-used the WiFi map surveyed during our earlier research presented in [4]. The type of the $d_{\mathrm{wifi}}$ measure and *k* used in WKNN was determined prior to indoor localization by comparing the accuracy of WiFi fingerprinting with an exhaustive set of different parameter settings on an independent training set. The best performance was reported for $\ell_{2}$ norm (the Euclidean norm of errors) with k=4. These parameters remained unchanged when compared to our previous work in the same environment [4].

Although a more accurate WiFi-based position estimate could be achieved with more accurate WiFi map or with algorithms that model the propagation of the signal in the environment [43], such an approach was not in the focus of this research. The WiFi scans are already not available on the iOS platform and soon the scanning rate on the Android devices will not be sufficient to properly run indoor localization solutions just on PDR and WiFi-based position estimates, regardless of the method used to compute these estimates. Therefore, we intentionally do not change our WiFi localization, focusing on the integration of visual information, and demonstrating that it is possible to localize a smartphone user even with a poor WiFi map, leveraging the qualitative visual information.

## 5. VPR

VPR focuses on estimating the user location based on the taken image. In our previous work [6], we focused on applying the well-known FAB-MAP algorithm [30] that is used in the robotic community to detect if the robot re-visits an already known location. We experienced several issues with the assumed approach:

- a single image does not provide enough information to obtain a successful indoor localization,
- even with correct recognition it is impossible to determine the metric localization due to scale ambiguity when performing 2D-2D image matching.

In [6], we solved the first problem by performing the same place recognition on several, consecutive images. In the same work, the second issue was solved by providing a weak topological constraint or a stronger constraint when an object of known size was present in the image or when the user provided the system with more accurate distance estimate. In the end, the resulting system turned out to be impractical, as the weak constraints were mostly useless for localization, while the stronger ones required too complicated interaction with the user. Thus, whereas the approach from [6] proved that it is possible to include non-metric constraints in the graph-based indoor localization framework, it was infeasible for implementation in a localization system intended for non-expert users.

In this article, we propose to directly focus on comparing sequences of recorded images that are greatly reduced to capture only the most important aspects of the image. The proposed solution is based on the FastABLE [35] algorithm that is a faster modification of the original ABLE-M/OpenABLE solution [44]. As all these VPR systems informed only about the visual similarity, we introduce a procedure that treats the matching images similarly as the matching WiFi scans are used in the WKNN algorithm. Finally, this procedure produces metric localization constraints that can be integrated into our graph-based estimation framework.

### 5.1. FastABLE Algorithm

**Figure 3:**
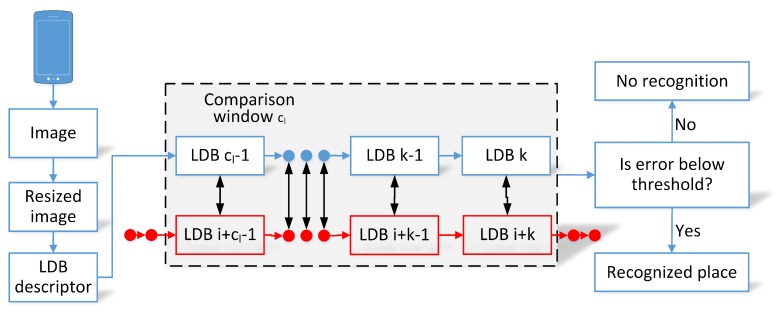
Processing pipeline of the FastABLE algorithm starts with Local Difference Binary (LDB) description of the captured image. The moving window of descriptors is compared to the window of a fragment of the map (sequence of images) to achieve an error measure. If the error is below the threshold, the correct recognition is reported.

The full processing of the FastABLE algorithm is presented in Figure 3. At first, each captured image is greatly resized to 64×64 pixels and then the LDB descriptor [45] for the whole image is computed. The descriptor is used to capture the visual characteristic of the image as a binary string.

As an LDB descriptor of a single image is not unique enough, the descriptors for consecutive images in a moving window are compared. The current images are compared to the pre-recorded sequences in the environment that are also stored as sequences of binary LDB descriptors. VPR is performed by comparing the training sequence of descriptors and the current sequence of descriptors in a moving window of size $c_{l}$ with the Hamming distance:

(9) $$d\left(a^{i},b^{j}\right)=\sum_{k=0}^{c_{l}-1}d_{\mathrm{hamming}}\left(a^{i-k},b^{j-k}\right),$$

where $a^{i}$ stands for the *i*-th image in the sequence *a*, and $d_{\mathrm{hamming}}\left(a^{i},b^{j}\right)$ denotes the sum of differences between the LDB descriptors in the moving window from the *i*-th position in the *a* sequence and the *j*-th position in the *b* sequence. The original algorithms (OpenABLE and FastABLE) determine the most similar matches to the chosen moving window that can be compared to some preset threshold but do not provide metric localization information.

Both algorithms greatly depend on temporal relations between consecutive images reducing the dependence on a single image. As a result, it is possible to greatly reduce the size of individual images providing the VPR system with additional robustness to image acquisition problems or small changes in the environment. Drastic changes in the environment or crowds in front of the camera might prevent the system from correctly recognizing the place but even in these conditions satisfactory results were reported as shown in [35].

### 5.2. Proposed Modifications to Obtain Localization Estimate

The FastABLE algorithm analyzes several consecutive images to determine visual similarity and thus will not operate properly on crossings of the corridors inside buildings. Therefore, the existing map of the environment is divided into *n* training sequences that cover the whole building assuming that sequences should not overlap in the same direction. Following the idea in [35], the automatic threshold computation is performed. In this process, each training sequence is matched against the remaining n−1 training sequences and the recognition threshold $t_{r}$ is set to the minimal value that results in no matches. This assumption is based on the fact that each sequence represents a different part of the environment and should not be matched by the system. These steps provide an initial recognition threshold for each sequence that is further multiplied by the coefficient $s_{r}$ that was determined experimentally and set to $s_{r}=1.2$.

**Figure 4:**
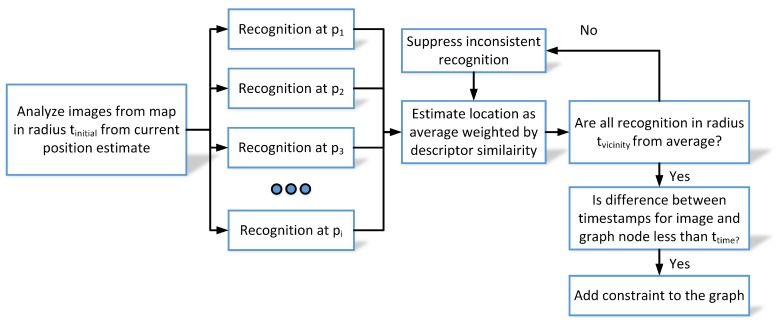
The processing starts with multiple matches with the FastABLE that reported the error below recognition threshold $t_{r}$ in a radius of $t_{\mathrm{initial}}$ from the current position estimate. The final estimate is determined by averaging the locations corresponding to these matches with weights determined as the inverse of the descriptor errors. Additional consistency checks (against $t_{\mathrm{vicinity}}$ and $t_{\mathrm{time}}$) are added to ensure reliable estimation and to prevent incorrect results.

The original FastABLE system informs that the user observes a part of the environment but fails to inform about the current orientation or exact metric location of the user. Therefore, we extend the system to obtain a more accurate position of the query image estimated upon the positions of the most similar source images, assuming that the positions of source images are stored in the environment map. This concept is presented in Figure 4.

During place recognition, the FastABLE matches the current window of last $c_{l}$ descriptors to all possible windows of descriptors in all training sequences and returns the list of matching places with the corresponding descriptor matching errors (the smaller the error, the better is the match). In order to obtain a system that operates correctly also in case of a self-similar environment, additional locality check was introduced that constrains the visual matches to a vicinity of the current pose estimate. In the presented version, the matches in a radius of $t_{\mathrm{initial}}=7$ m from the current location estimate were accepted for further processing.

For each match, the place recognition estimate $p_{\mathrm{pvr}}$, is computed as the weighted average of locations of all matches with errors smaller then thresholds:

(10) $$p_{\mathrm{vpr}}=\frac{\sum_{i=1}^{k}s\left(a,b_{i}\right)p_{i}}{\sum_{i=1}^{k}s\left(a,b_{i}\right)},$$

where *a* stands for the currently analyzed window of descriptors, $b_{i}$ is the window of descriptors for the *i*-th match below threshold and $s(a,b_{i})$ is the similarity measure between these windows. The *k* determines the number of matches below the recognition threshold while $p_{i}$ is the location of the *i*-match. The similarity of two sequences is set as the inverse of the sum of the Hamming distances between corresponding descriptors for sequences:

(11) $$s\left(a,b_{i}\right)=\frac{1}{d_{\mathrm{hamming}}\left(a,b_{i}\right)+eps},$$

where eps is a small value to ensure proper computation in the unlikely scenario of $d_{\mathrm{hamming}}\left(a,b_{i}\right)=0$.

The final place recognition estimate is obtained as a weighted average of all accepted recognition and thus is susceptible to outliers. Thus, we propose further processing that rejects any match if its position $p_{i}$ is estimated to be further than $t_{\mathrm{vicinity}}=3$ m from the VPR estimate $p_{\mathrm{vpr}}$. In that case, this recognition is ignored and the whole estimation is repeated for the remaining matches. This procedure, inspired by the WKNN algorithm for WiFi scans, provides a better estimate of the query image location than matching to just a single image from the known map (sequence of images). The location of the query image is assumed to be the location of the user holding the smartphone’s camera.

### 5.3. VPR as Graph-Based Constraint

The proposed VPR system provides exact position estimates based on the captured images and therefore it is possible to represent the measurement as a graph edge. With the current estimate of the user’s pose $v_{i}$ and VPR’s result $p_{\mathrm{vpr}}=\left(x_{\mathrm{vpr}},y_{\mathrm{vpr}}\right)$, the error of the edge can be defined as:

(12) $$e_{i}^{\mathrm{vpr}}=\left[\begin{array}{c} x_{i}-x_{\mathrm{vpr}}\\ y_{i}-y_{\mathrm{vpr}} \end{array}\right].$$

In the proposed version of the error, the correct VPR does not constrain the angle of the observation since sequences can be matched from a range of views. Nevertheless, the exact orientation could be computed using the n-point algorithm [46]. This procedure has been shown to be feasible on a smartphone in our earlier work [27] but is not used here because the pre-surveyed sequences of the visual map do not have accurate orientation information. Additionally, the n-point algorithm requires distinct visual features that are hard to detect in a self-similar, corridor-like environment. Hence, the n-point algorithm, being quite expensive in terms of computations, does not bring any useful orientation estimate with respect to the trajectory taken by the user.

**Figure 5:**
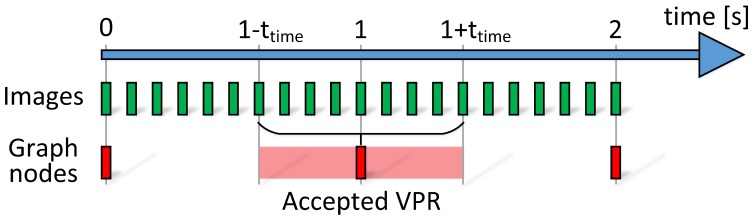
The images are processed with greater frequency than the nodes representing user position are placed in the graph. Therefore, the VPR is accepted for a graph node when the difference between timestamps of an image and a graph node is below $t_{\mathrm{time}}$ = 0.5 s.

As the images from the camera are recorded with 10 Hz, the VPR estimates can be determined with the same frequency. On the other hand, the nodes in the graph representing the user locations are placed with a frequency of about 1 Hz that is significantly lower than the frequency of possible matches from the VPR. The mentioned problem is visualized in Figure 5. This problem could be solved by increasing the number of graph nodes but it would also increase the computational burden of optimization due to the increased number of states to estimate. We propose that each VPR estimate is introduced to the graph if the difference between the timestamp of the image and the timestamp of the selected node in the graph is below the $t_{\mathrm{time}}$ = 0.5 s. The value of 0.5 s was chosen as a tradeoff between the inaccuracies introduced due to the incorrect timestamps and the number of states estimated in the optimization.

The information matrix of the VPR system is set to $\Omega^{\mathrm{vpr}}=k^{\mathrm{vpr}}I_{1\times 1}$, with $k^{\mathrm{vpr}}$ experimentally set to 10.

### 5.4. Visual Map Acquisition and Storage

**Figure 6:**
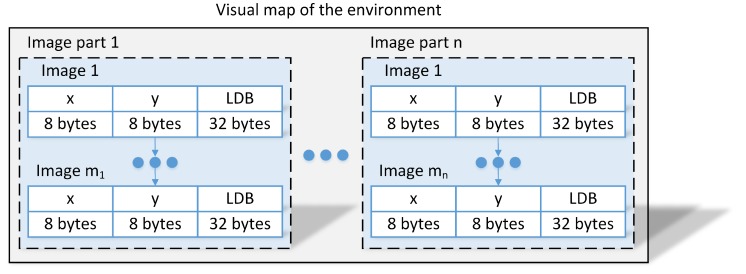
The visual map of the environment consists of multiple parts for each independent route in the environment. Each part consists of a sequence of images stored as memory-efficient LDB descriptors with corresponding absolute positions.

The considered application of the VPR algorithm requires a pre-surveyed map of the environment that is used when comparing the images taken during the localization to known locations. The usual visual map consists of multiple parts with each part covering a single route without crossroads in the environment. Each part is comprised of consecutive images (image sequences) while moving along the possible route with a reference absolute location for each image. Each image in the visual map is preprocessed and stored as a 256-bit (32 bytes) global descriptor instead of images providing a concise representation of the environment with corresponding (x,y) positions (16 bytes) with respect to the global frame as presented in Figure 6. This representation could easily be extended to include additional information about, i.e., floor number or even reduced with additional assumptions about relations between locations of images in a single part.

Whenever the visual map is acquired using a smartphone, the reference locations for captured images with respect to the global frame are needed. We obtained these coordinates by assuming the constant velocity of the motion with the beginning and end of each sequence anchored to known, characteristic positions in the floor plan of the building. The characteristic positions are chosen manually, in our test environment they are corridor junctions, dead ends or fire doors that divide the corridor. Although any other localization system (e.g., motion capture equipment or a separate camera and laptop for visual odometry) can be used to obtain the reference locations, we consider the use of a smartphone as a practical, low-cost solution that yields positions of images that are accurate enough with respect to the known floor plan.

**Figure 7:**
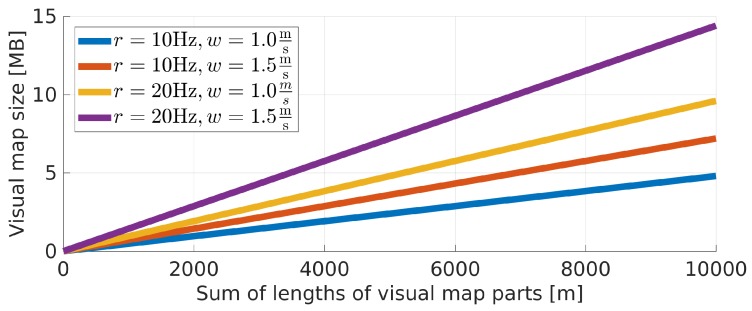
Dependence between the visual map size and the total length of this visual map parts shown for selected values of the image capture rate *r* and walking speed *w*.

Preprocessing the original images provides a concise representation of the environment that can easily be stored even for large buildings on a mobile device. Figure 7 presents a simulation of a map size depending on the metric length of all parts of the visual map with the assumed image capture rate *r* and walking speed *w*. Even with r=20 Hz and $w=1.0\frac{m}{s}$ the map size did not exceed 15 MB for 10 km of routes in the environment making it feasible to deploy in real-life scenarios. It is important to note that the images should be captured at a similar frequency and similar walking speed when compared to sequences captured during the localization. We observed no issues with slight deviations of walking speeds. Nevertheless, care should be taken to measure the user’s speed and adjust data in a visual map to match the user’s speed and image capture rate.

## 6. Experimental Evaluation

### 6.1. Experimental Setup

The experimental verification requires additional image information that is not commonly recorded for indoor localization datasets and was also not captured in our previous works. Therefore, a new dataset was recorded. In our experiments, a user equipped with the horizontally-held, front-facing smartphone was asked to continuously move around the University building called PUT MC along several trajectories. The Android-based system captured the raw data that was further processed with the code that is made publicly available at Github (https://github.com/LRMPUT/IndoorGraphLocalization in branch *vpr*).

**Figure 8:**
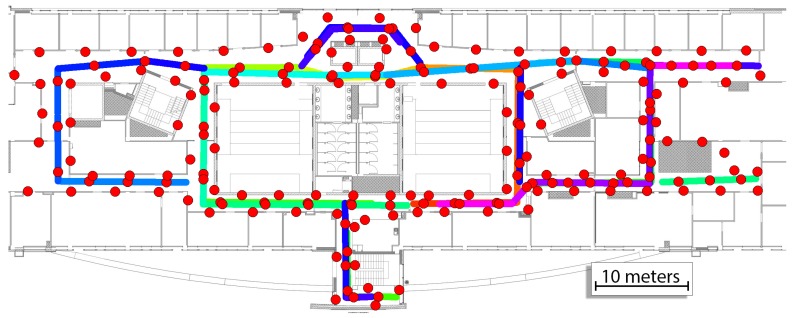
The above map of the environment contains information about the WiFi scans (**red circles**) and map parts containing images recorded when walking in both directions (**lines with a different color for each segment**).

The prior map for the system consists of the WiFi map and image map for VPR. The WiFi map was taken from our previous work [4]. The whole image map was divided into 26 map parts and sequences for VPR were captured in both directions for all available corridors. The WiFi and image maps are presented in Figure 8. Each image taken in the training sequence has an assumed location that was estimated based on the movement in the environment with the assumption of constant velocity between points annotated on the map. The total number of images in the map is equal to 3489 images with the exact size of each part presented in Table 1.

Some of the recorded image parts are short and therefore contain a small number of images. These parts might not be used in the processing if the comparison window $c_{l}$ in the FastABLE is set to the value greater than the length of that sequence due to an insufficient number of images that are needed for the computation of bluean error.

As the information from images is already precomputed, each image is stored as a single 256-bit descriptor and thus the whole map for VPR takes around 0.85 MB in memory. The LDB descriptors for each map part are also used to automatically determine the safety detection thresholds for each map part that are determined prior to real-life operation.

**Figure 9:**
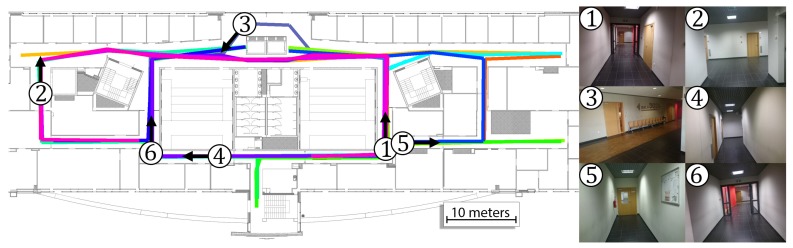
The ground truth motion for 14 trajectories in the environment with each trajectory marked by a different color and six exemplary views from the environment.

The performance of the system was verified on 14 sequences that were recorded by three users with the same Xperia Z3 smartphone. The users moved in the environment on paths that resembled real motion. The length and the number of images for each sequence are presented in Table 2. The total length of all sequences is equal to 1025.4 m, and the total number of captured images is equal to 8960. The ground truth motion for these sequences was manually annotated on blueprints of the building and is presented in Figure 9. With this approach, there is no possibility to measure the user’s location error at the selected timestamp and the error is computed as a distance between the estimated user pose and the closest point that lies on the ground truth trajectory. The error for the selected sequence is computed as the RMSE or average error of all positions estimated by the indoor localization system.

### 6.2. Performance of the System without VPR

**Figure 10:**
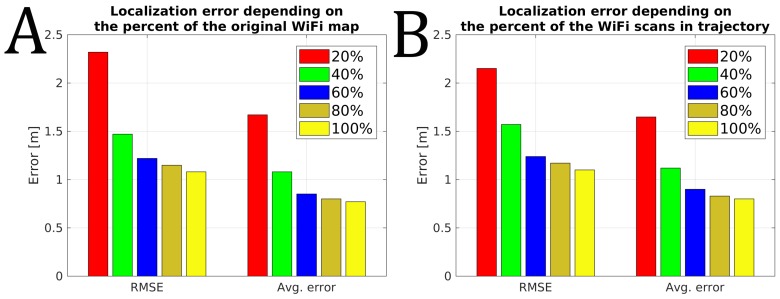
The performance of the original system depending on the percentage of the original WiFi map size (**A**) and the percentage of processed WiFi scans from trajectories (**B**).

The existing infrastructure-free, smartphone-based indoor localization solutions rely heavily on WiFi signals to provide accurate absolute localization. The recent changes in the Android WiFi scanning policy reduce the allowed frequency of WiFi scanning that influences the performance of such systems. We wanted to measure the performance of the proposed system without VPR with the reduced ability to rely on WiFi scans. Therefore, we measured the indoor localization accuracy when the frequency of the WiFi scans was reduced and when the map of the environment degrades over time. The obtained results for our trajectories are presented in Figure 10.

**Figure 11:**
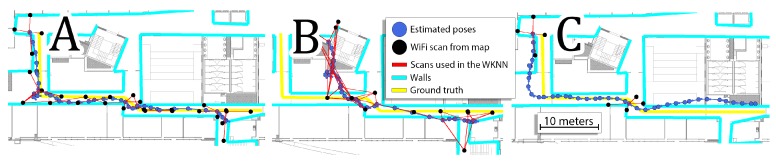
Exemplary result obtained for sequence 4 when: all WiFi information was used (**A**), only 20% of the original WiFi map was used (**B**), and only every 5-th of the WiFi scans from the acquired sequence was used (**C**).

Reducing the amount of WiFi information available for the indoor localization system results in an increased localization error. When all available information is used, the system reports the RMSE of about 1.1 m and an average error of 0.8 m when averaged for 14 available sequences. This accuracy degrades even to 2.3 m of RMSE error when only 20% of the original pre-surveyed WiFi map is used and it could be even worse as this value was obtained by averaging several runs with a random selection of WiFi scans from the original map. A similar negative influence is observed when a dense WiFi map is available, but only every 5-th WiFi scan from those acquired during the localization experiment is used. This limitation, directly modeling a limit set on the WiFi scanning frequency, resulted in RMSE of 2.15 m. The two scenarios of WiFi data degradation presented above result in similar localization errors, but they represent two different issues that a smartphone-based indoor localization system can face in practice. The former one simulates the negative influence of time passed from the moment the map has been surveyed, as during that time the constellation of WiFi networks in the given environment could change, thus the number of detectable networks could decrease. The latter case simulates a situation when the WiFi scans cannot be acquired as often as necessary, e.g., due to the smartphone’s operating system limitations. Both cases present different challenges to localization, as presented in Figure 11.

Random selection of a part of the dense WiFi map results in reporting locations that are far away from the true position of the user, thus introducing a strong bias to the estimated trajectory. This problem is typically solved by updating the WiFi map by surveying the environment again or by adding new scans from networks discovered online, like in our recent work [4]. On the other hand, processing only a subset of available WiFi scans from the sequence results in sparse absolute localization requiring the PDR to fill in the missing parts of the trajectory only upon the data from inertial sensors. This is a problem that we are currently faced with less frequent WiFi scans on the Android platform, but one should remember that the much reduced WiFi scanning frequency makes it also problematic to improve a degraded global WiFi map. Therefore, this article presents how VPR can be used to overcome both of these challenges.

### 6.3. Performance of the System with VPR

The presentation of the performance of the system utilizing PDR and WiFi scans without VPR in a previous section serves as a basis for further analysis. Therefore, the left part of Table 3 contains the results obtained for 14 considered sequences in that configuration. In this experiment, the original performance of the system is compared to the system with additional VPR information. The results for this configuration are presented in the right part of Table 3. The performance of the VPR depends on several parameters ($c_{l}$, $t_{\mathrm{recognition}}$, $t_{\mathrm{vicinity}}$, $t_{\mathrm{initial}}$, $t_{\mathrm{time}}$) that were determined experimentally and are the same for all sequences and further analysis. It is probably possible to further tune the parameters of the system but we wanted to avoid overfitting the parameters to our sequences.

**Figure 12:**
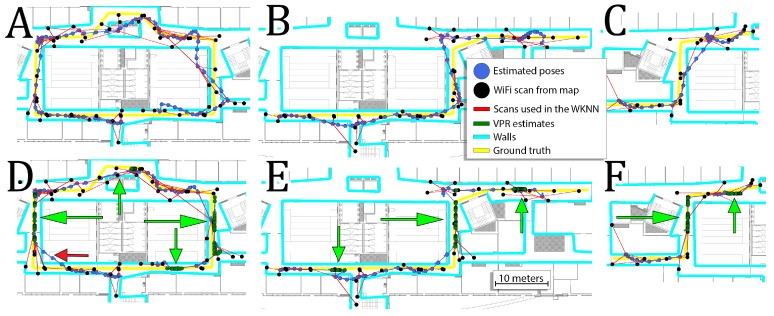
Comparison between trajectories obtained without (**A**–**C**) and with VPR (**D**–**F**) for sequences 1 (**A**,**D**), 9 (**B**,**E**), and 12 (**C**,**F**). Green arrows highlight the parts of the trajectories that were corrected by the VPR information while red arrow presents the negative impact of the VPR.

Additional information about the VPR reduces the average RMSE for all sequences by about 13% and average error for all sequences by about 20%. Despite the overall reduction, the additional VPR constraints can sometimes have zero or even negative effect on the obtained performances on some sequences. This is mostly caused by the fact that the VPR results are not evenly distributed for the whole lengths of the sequences. For locations with VPR constraints, the error is usually significantly reduced but it may have a negative impact on other parts of the sequences. The trajectories obtained for sequences 1, 9, and 12 with and without VPR are presented in Figure 12. In most cases, the VPR constraints reduce the trajectory error and can even provide almost perfect localization for several meters. In some cases, the VPR provides an additional source of information that can correct inaccurate WiFi localization. Unfortunately, the VPR does not help on the crossing when additional challenges must be faced due to large orientation changes.

**Figure 13:**
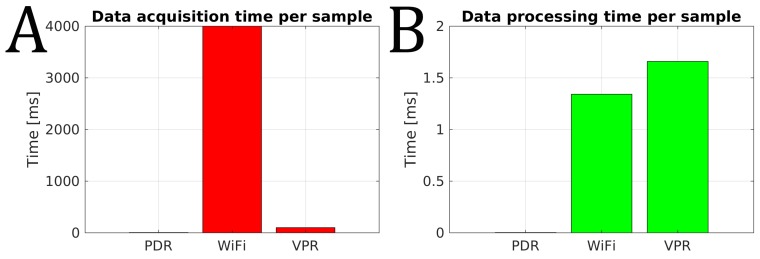
Comparison between the time required to capture and process data by PDR, WiFi fingerprinting, and VPR: data acquisition time (**A**) and data processing time (**B**). Note that all measured times are in ms. These times are different on different devices, but the proportions remain largely unchanged.

Commonly, the image processing algorithms are considered time-consuming and generating additional localization latency. In order to show that this is not the case for our solution, we measured and present the acquisition and processing times for the three main components of our positioning system (Figure 13).

The data acquisition for PDR is performed with 200 Hz from accelerometers, while a single WiFi scan takes around 4 s. The camera images are captured at 10 Hz, so we can safely assume that the visual latency is not greater than 100 ms at this stage, and is much smaller than for the WiFi fingerprinting component (Figure 13A). The PDR data sample processing is fast with FFT taking significantly less than 1 ms. The processing time for WiFi fingerprinting depends on the size of the WiFi map (number of fingerprints), and it took 1.34 ms on average to process a single WiFi scan for the map used in our experiments. The single image processing is fast, taking 1.66 ms on average (Figure 13B), which is a result of several factors: resizing the images to low resolution, fast binary descriptor comparison, and reusing the previous VPR results with the FastABLE algorithm performing descriptor comparisons in a moving window [35]. Hence, the processing time for images is comparable with the same parameter for the WiFi scans, and the whole place recognition system runs very fast in comparison to a classic visual odometry algorithm implemented on a mobile device [27]. Taking into account both the acquisition time and the processing time we can conclude that the WiFi fingerprinting module provides position estimates at 0.25 Hz, while VPR can provide comparable estimates with the frequency of 10 Hz.

### 6.4. VPR with Sparse WiFi Map

With the reduced amount of WiFi scans on the map, we would like to measure the robustness of the system. In this experiment, the system without VPR information was compared to the system with VPR information. The results for every execution are averaged by 10 runs and are presented in Table 4.

**Figure 14:**
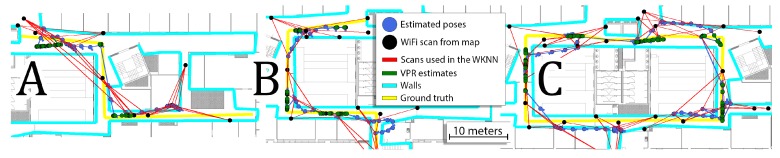
Trajectories obtained for sequences 5 (**A**), 7 (**B**), 10 (**C**) when only 20% of the original WiFi map is used. The usage of VPR makes is possible to correct trajectories despite inaccurate WiFi measurements.

Independently of the kept percent of the WiFi map, the information from the VPR reduces the RMSE and the average error. The gain from the VPR is more evident when the percentage of the original WiFi map is lower, due to the fact that the WiFi information is more inaccurate. For these conditions, the VPR information is more important than when the full WiFi map is available. The gains from the VPR are between 10% and 30%. Although the robustness of the localization system has been increased, it still requires a pre-surveyed WiFi map for proper operation. Figure 14 presents trajectories obtained for sequences 5, 7 and 10 when only 20% of the original WiFi map were used.

On these trajectories, we can see that the VPR is crucial in obtaining accurate estimates as WiFi localization is mostly greatly inaccurate. The VPR corrects trajectory when it is available, but when VPR is not recognized, the system is inaccurate due to poor WiFi-based localization. Moreover, when the WiFi map is very sparse, incorrect matches of WiFi scans occur frequently. Eventually, inconsistent estimates from the VPR and WiFi WKNN result in trajectories that are not locally smooth.

### 6.5. VPR with a Reduced Number of WiFi Scans in Trajectory

The current Android WiFi scanning policy reduces the maximum WiFi scanning frequency and therefore we are interested in verifying the performance of the system with the reduced number of processed WiFi scans for each sequence. This comparison is performed without and with VPR to measure the possibility of applying the created system in the current Android environment. The obtained results averaged on all analyzed sequences are presented in Table 5.

The processing only a part of available WiFi scans makes the system more dependent on the estimates from the PDR to provide a sensible trajectory estimate. Therefore, the addition of the VPR almost universally decreases the localization error when compared to the version without VPR. Naturally, with longer gaps between WiFi scans system is susceptible to drift. This prevents the VPR from improving the performance of the system when only 20% of the available WiFi scans are processed. This can be expected, as VPR determines its matches in local surroundings due to the environment self-similarity, and might yield suboptimal matches between images when the current estimate is far from the user’s real position in the environment. However, the VPR constraints were correct even in those cases, but the estimated trajectory was prone to drift due to the high dependency on the PDR, and large distances between the “anchors” provided by the few available constraints. When the percentage of the pre-surveyed WiFi scans was greater than 20%, the improvement varied from 10% to 20% when VPR was used. What is interesting, the system achieves almost the same accuracy with the use of 60% WiFi scans for the trajectory and with VPR, as in the case when the VPR matches were not considered, but all of the available WiFi scans were used.

**Figure 15:**
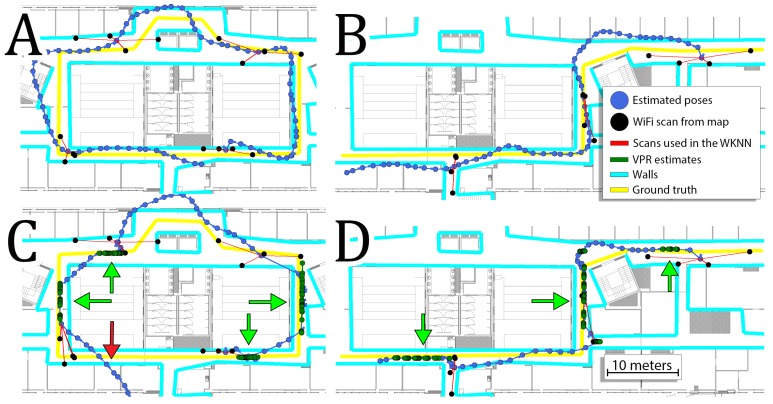
Comparison between trajectories obtained without (**A**,**B**) and with VPR (**C**,**D**) for sequences 1 (**A**,**C**) and 9 (**B**,**D**) when 20% of WiFi scans for each trajectory were used. **Green arrows** point to the parts of trajectory that were corrected due to VPR, while the **red arrow** shows the negative effect that is amplified by the reliance on the PDR.

The exemplary trajectories obtained in that experiment are presented in Figure 15. The smaller number of available WiFi scans results in a trajectory that roughly follows the real motion of the user as presented in Figure 15A,B. This trajectory depends on PDR for long periods of time. The addition of the VPR constraints makes it possible to reduce the error when WiFi information is not available. However, even a correct position constraint from the VPR might unintentionally change the orientation estimate, causing that the PDR follows the trajectory in the wrong direction (Figure 15). In this extreme case, the localization system requires more information (denser WiFi scans or more images in the visual map) to obtain a better position estimate.

### 6.6. VPR as WiFi Alternative

**Figure 16:**
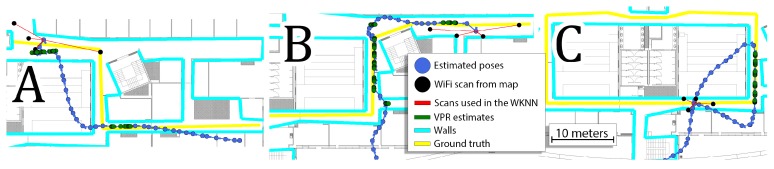
The trajectories obtained with a system processing PDR and VPR constraints with a single, initial location from WiFi on sequences 5 (**A**), 9 (**B**), and 13 (**C**). The obtained VPR are correct but are too sparsely detected in the environment, which sooner or later leads to incorrect estimates.

The goal of this experiment is to answer the question if the VPR in the proposed version can substitute the WiFi information altogether. Therefore, for each sequence, only the first WiFi scan from the acquired test sequence was used to determine the initial position of the user, and then the localization system relied on the PDR and VPR to provide position estimates. The obtained errors were significantly larger than in any of the previously considered cases, and thus we propose to analyze selected trajectories presented in Figure 16.

The results obtained in the presented sequence show that the detected VPR matches are correct, but are too sparsely detected in the environment to provide an accurate position estimate of the user. In every analyzed case, the system sooner or later diverges from the real path of motion. Once the local estimate is too far from the real position, the VPR no longer searches for matching images in the proper neighborhood, which in turn causes the PDR to drift away from the true trajectory. This demonstrates that while the VPR is suitable as an additional source of localization constraints, it cannot be used without WiFi WKNN constraints in the current form. Better performance could probably be expected in an environment with fewer self-similarities, but still, the WiFi-based localization can be considered more robust than the VPR.

## 7. Conclusions

The article presents a way of utilizing the VPR for indoor localization and its integration into the existing indoor localization framework. The VPR is performed based on a sequence of whole-image LDB descriptors that capture the information stored in the images captured by a smartphone’s camera. The usage of sequences allows our visual localization subsystem to operate in a highly self-similar environment, while further modifications to the FastABLE algorithm proposed in this paper make it possible to obtain an accurate position estimate of the user with respect to a known map of images. The proposed procedure, inspired by the WKNN algorithm used to process the matching WiFi scans, yields metric position information which is then introduced as constraints to the graph-based localization framework. The form of metric position estimates computed by our VPR method is also compatible with particle filtering and Kalman filtering frameworks, hence its ability to be adapted to other indoor positioning systems.

The experiments were performed inside one of our University’s buildings with multiple visually self-similar corridors. In spite of these similarities, the test locations were correctly distinguished by the system. Additional VPR improves the performance of the system operating on PDR and WiFi scans. The experiments with selected percentages of the original WiFi map reveal that the addition of VPR makes the localization system more robust, as it can still operate despite inaccurate WiFi-based position estimates. In the case of a reduced number of WiFi scans that are acquired by the user while he/she uses the localization system, the configuration with VPR presents improved accuracy and robustness. Nevertheless, the experiments without WiFi scans reveal that the VPR constraints alone do not provide enough information to prevent the estimated trajectory from a significant drift. This problem is related to the local search policy of the place recognition algorithm: Incorrect matches lead to a trajectory drift, while the trajectory drift prevents the algorithm from finding the correctly matching images. On the other hand, this local policy is necessary to prevent the VPR algorithm from matching the query images to locations that are far away but are visually similar due to the nature of the office-like environment. Therefore, despite certain gains from VPR, in the presented form, it cannot replace WiFi signals. The code of the solution is publicly available, making it possible to verify our results and build on our experience.

## Figures and Tables

**Table 1:** The environment was divided into 26 image sequences that capture all main routes in both directions.

Map part	0	1	2	3	4	5	6	7	8	9	10	11	12
No. images	101	107	80	31	47	36	77	119	218	201	97	70	360
Map part	13	14	15	16	17	18	19	20	21	22	23	24	25
No. images	122	103	52	156	202	102	73	445	258	129	107	35	161

**Table 2:** Lengths of the recorded sequences. The total length of all recorded sequences is equal to 1025.4 m and the total number of captured images is equal to 8960.

Sequence	1	2	3	4	5	6	7
Length [m]	101.6	49.09	79.1	50.49	53.11	52.66	49.72
No. images	864	456	604	458	454	454	411
Sequence	8	9	10	11	12	13	14
Length [m]	109.39	73.33	97.69	67.66	39.63	100.96	100.97
No. images	888	615	857	687	331	826	1055

**Table 3:** Comparison between reported accuracy for analyzed sequences for the original configuration of the system (PDR + WiFi) and the configuration with additional VPR (PDR + WiFi + VPR).

Localization Error of PDR + WiFi						Localization Error of PDR + WiFi + VPR					
Seq.	**RMSE**	**Avg. Err.**	Seq.	**RMSE**	**Avg. Err.**	Seq.	**RMSE**	**Avg. Err.**	Seq.	**RMSE**	**Avg. Err.**
1	1.16	0.81	8	1.29	0.86	1	0.72	0.55	8	1.20	0.75
2	0.89	0.7	9	1.25	0.94	2	0.82	0.62	9	1.16	0.79
3	0.98	0.74	10	1.22	0.92	3	1.19	0.60	10	1.05	0.67
4	0.76	0.61	11	1.39	0.84	4	0.71	0.53	11	1.06	0.65
5	0.98	0.78	12	0.94	0.68	5	0.98	0.80	12	0.47	0.40
6	0.52	0.39	13	1.03	0.82	6	0.41	0.29	13	0.83	0.54
7	1.11	0.85	14	1.07	0.84	7	0.92	0.66	14	0.99	0.78
All	1.1	0.8				All	0.96	0.64			

**Table 4:** The comparison between the accuracy of the system with and without VPR with different percent of the original WiFi map for 14 trajectories averaged over 10 runs.

	Percentage of the Original WiFi Scans in the WiFi Map									
	**20%**		**40%**		**60%**		**80%**		**100%**	
**VPR**	**RMSE**	**Avg. Err.**	**RMSE**	**Avg. Err.**	**RMSE**	**Avg. Err.**	**RMSE**	**Avg. Err.**	**RMSE**	**Avg. Err.**
Off	2.32	1.67	1.47	1.22	1.22	0.85	1.15	0.8	1.1	0.8
On	1.76	1.17	1.29	0.83	1.06	0.69	1.02	0.67	0.96	0.64
Gain	24.1%	29.9%	12.2%	23.2%	13.1%	18.8%	11.3%	16.3%	12.7%	20%

**Table 5:** The comparison between the accuracy of the system with and without VPR with different percent of the original WiFi scans for 14 trajectories averaged over 10 runs.

	Percentage of the Original WiFi Scans in the Trajectory									
	**20%**		**40%**		**60%**		**80%**		**100%**	
**VPR**	**RMSE**	**Avg. Err.**	**RMSE**	**Avg. Err.**	**RMSE**	**Avg. Err.**	**RMSE**	**Avg. Err.**	**RMSE**	**Avg. Err.**
Off	2.15	1.65	1.57	1.12	1.24	0.9	1.17	0.83	1.1	0.8
On	2.99	2.0	1.34	0.86	1.07	0.72	0.99	0.65	0.96	0.64
Gain	-	-	14.7%	23.2%	13.7%	20.0%	15.4%	21.7%	12.7%	20%
